# Investigations on the mode of action of gephyronic acid, an inhibitor of eukaryotic protein translation from myxobacteria

**DOI:** 10.1371/journal.pone.0201605

**Published:** 2018-07-31

**Authors:** Yazh Muthukumar, Johanna Münkemer, Daniel Mathieu, Christian Richter, Harald Schwalbe, Heinrich Steinmetz, Wolfgang Kessler, Joachim Reichelt, Ulrike Beutling, Ronald Frank, Konrad Büssow, Joop van den Heuvel, Mark Brönstrup, Richard E. Taylor, Sabine Laschat, Florenz Sasse

**Affiliations:** 1 Department of Chemical Biology, Helmholtz Centre for Infection Research, Braunschweig, Germany; 2 Institut für Organische Chemie, Universität Stuttgart, Stuttgart, Germany; 3 Zentrum für Biomolekulare Magnetische Resonanz, Universität Frankfurt, Frankfurt, Germany; 4 Department of Microbial Drugs, Helmholtz Centre for Infection Research, Braunschweig, Germany; 5 Department of Structure and Function of Proteins, Helmholtz Centre for Infection Research, Braunschweig, Germany; 6 Department of Chemistry & Biochemistry, University of Notre Dame, Notre Dame, Indiana, United States of America; Wayne State University, UNITED STATES

## Abstract

The identification of inhibitors of eukaryotic protein biosynthesis, which are targeting single translation factors, is highly demanded. Here we report on a small molecule inhibitor, gephyronic acid, isolated from the myxobacterium *Archangium gephyra* that inhibits growth of transformed mammalian cell lines in the nM range. In direct comparison, primary human fibroblasts were shown to be less sensitive to toxic effects of gephyronic acid than cancer-derived cells. Gephyronic acid is targeting the protein translation system. Experiments with IRES dual luciferase reporter assays identified it as an inhibitor of the translation initiation. DARTs approaches, co-localization studies and pull-down assays indicate that the binding partner could be the eukaryotic initiation factor 2 subunit alpha (eIF2α). Gephyronic acid seems to have a different mode of action than the structurally related polyketides tedanolide, myriaporone, and pederin and is a valuable tool for investigating the eukaryotic translation system. Because cancer derived cells were found to be especially sensitive, gephyronic acid could potentially find use as a drug candidate.

## Introduction

The bacterial translation system is well characterized due to the availability of a wide range of small molecule prokaryotic translation inhibitors and high-resolution crystal structures. In contrast, only a few translation inhibitors are known for eukaryotes. Therefore, there is a dire need for the identification of new compounds inhibiting eukaryotic translation [1–3]. Given the crucial role of protein biosynthesis in cancerous cells, eukaryotic translation inhibitors are potentially of great importance for the treatment of cancer [4, 5].

**Fig 1:**
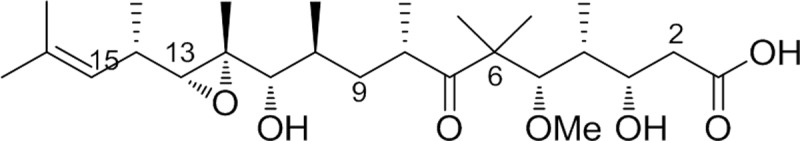
The chemical structure of gephyronic acid 1.

Gephyronic acid (GA; Fig 1), isolated from the myxobacterium *Archangium gephyra*, was first reported from our laboratory in 1995 [6]. It had strong anti-proliferative activity on mammalian cells and fungi but showed no effects on prokaryotes. The chemical structure was later revised and confirmed by total synthesis [7, 8]. It resembles myriaporone, the southern hemisphere of tedanolide, and forms a structural link to pederin. All these related polyketides are known to inhibit protein synthesis [9].

## Materials and methods

### Gephyronic acid

Gephyronic acid (GA) was produced by fermentation of the myxobacterial strain *Archangium gephyra* Ar 3895. The fermentation and isolation procedure has been published [6].

### Cell cultures

Cell lines were obtained from German Collection of Microorganisms and Cell Cultures (DSMZ) and the American Type Culture Collection (ATCC) and grown in culture media recommended by the supplier. Human dermal fibroblasts (NHDF) from Lonza were grown in FGM-2 supplemented with FGM-2 bullet kit (Lonza). All cell cultures were kept at 37°C and 10% CO_2_.

### Luminescence cell viability assay

The cytotoxic effects of GA on primary (NHDF) and cancer cells (KB-3-1) were measured using the CellTitre-Glo® (Promega). In brief, 10,000 cells/well were seeded in a 96-well plate, grown overnight and incubated with serial dilutions of GA ranging from 10 to 0.001 μg/mL. The final culture volume was 100 μL. After 48 hours, the cells were mixed with CellTitre-Glo®. The resulting luminescence was measured and related to controls with the vehicle only.

### In vitro translation inhibition assay

Translation inhibition assays were performed by using the Flexi Rabbit Reticulocyte Lysate system (Promega) or wheat germ extract (Promega). Briefly, 500 ng of luciferase control RNA encoding the firefly luciferase gene, which is provided with the kits, was combined with 17.5 μl of rabbit reticulocyte lysate, 0.25 μl each of 1mM–Met and–Leu amino acid mixtures, 0.7 μl of 2.5 M KCl, 10 U RNasin, 3.3 μl of nuclease free water and 1 μl of GA to give a final concentration ranging from 100 to 0.001 μg/ml. Reactions were incubated at 30°C for 90 minutes. Luminescence was measured by mixing 2 μl of the mixture with 10 μl of the luciferase assay substrate (Promega).

### Single luciferase reporter assay

100 μl DMEM medium containing 10^6^ KB-3-1 cells were seeded in a 96-well plate. After 16 hours, the cells were transfected with pRLSV40 vector (Promega) using PEI as a transfection agent. After 48 hours, cells were treated with GA at concentration ranges of 10 to 0.001 μg/mL for 6 hours. Following, the medium was removed and the cells were lysed with 30 μl of 1X Renilla luciferase assay lysis buffer (Promega). 10 μl of the lysate was mixed with 10 μl of 1X Renilla luciferase assay substrate (Promega), and the luminescence was measured.

### IRES dual luciferase reporter assay

The vectors pBClucPRluc (kind gift from Dr. Mario Köster, HZI) and pFR-CrPV-xb (Addgene plasmid 11509) [10] were transfected and treated as mentioned above. After 3 hours the cells were lysed with passive lysis buffer (Promega) and Renilla and firefly luminescence were measured using the dual luciferase reporter assay buffer (Promega).

### DARTS analysis

KB-3-1 cells cultured to 70–80% confluency were lysed with M-PER lysis buffer (Thermo Fisher Scientific) and the protein content was measured. 40 μg of the protein lysate was incubated with GA, cycloheximide or methanol for 2 hours at RT. The mixture was digested with 20 ng of Pronase (Roche) for 30 min at 37°C. The digestion was stopped by heating with 4X loading dye (Roth). The digest was separated by electrophoresis in 4–20% Tris-HCl gels and transferred onto a nitrocellulose membrane. The membrane was blocked with 5% BSA and probed with rabbit polyclonal antibodies against eIF2α and GAPDH (Cell Signalling). Anti-rabbit HRP conjugated antibodies (Cell signalling) were used as secondary antibodies and detected by chemiluminescence.

### Biotinylation of gephyronic acid

To a stirred solution of gephyronic acid methylester **2** (8.3 mg, 17.1 μmol) in CH_2_Cl_2_ (2 mL) were added D-(+)-biotin **3** (8.4 mg, 34.4 μmol), DCC (8.1 mg, 39.4 μmol) and DMAP (1.0 mg, 8.5 μmol) at room temperature. After stirring for 12 days, the reaction mixture was filtered through celite and the solvent was removed under vacuum. Purification by column chromatography on silica gel 60 (grain size 0.04–0.063 mm, Fluka) and ethyl acetate/MeOH (10:1) as eluent provided **4a** (0.8 mg, 1.1 μmol, 7%) and **4b** (6.0 mg, 8.5 μmol, 50%) as colorless amorphous solids. ^1^H and ^13^C NMR spectra were recorded on Bruker Avance 600 (600 MHz and 150 MHz, respectively) and Avance 800 (800 MHz and 200 MHz, respectively) spectrometers. ^13^C NMR multiplicities were determined with DEPT experiments. The regioisomeric (C-11)-biotinylated compound **4a** and (C-3)-biotinylated compound **4b** were characterized by COSY, ROESY, HSQC and HMBC experiments. FTIR spectra were recorded on a Bruker Vektor 22 spectrometer. Mass spectra were measured on a Bruker Daltonics microTOF-Q ESI mass spectrometer. Optical rotations were measured on a Perkin-Elmer Polarimeter 241. Gephyronic acid methylester **2** was prepared according to literature procedures [7]. D(+)-Biotin **3** was commercially available.

### Fluorescence staining

Overnight cultured PtK2 (ATCC CCL-56) cells were incubated with 50 nM GA or **4b** for 3 hours. Then the cells were fixed with formaldehyde (3.7%) for 10 min, incubated with mouse anti-eIF2α antibody (Santa Cruz Biotech) and with streptavidin that was labeled by Alexa Fluor 488 (Molecular Probes) for one hour, and counter stained with anti-mouse Alexa Fluor 594 antibodies. Cells were rinsed with PBS, mounted in Prolong Antifade (Molecular Probes), and viewed with a Zeiss Axiophot fluorescence microscope by using appropriate filter sets.

### Pull down assay with cells

70–80% confluent KB-3-1 cells were lysed with M-PER lysis buffer (Thermo Fisher Scientific) supplemented with 1x Halt™ protease and phosphatase inhibitor cocktail (Thermo Fisher Scientific). Following protein measurements by Bradford’s method, 200 μl aliquots containing 5 mg/ml of protein were incubated with 2 μl methanol, 2 μl GA (1 mg/ml), 2 μl biotinylated GA methyl ester (1 mg/ml), or 2 μl of a 1:1 mixture of both at 4°C for 1 hour. Following, the lysates were incubated with streptavidin coated beads for 1 hour at RT, washed and heated at 96°C with loading dye. After a SDS-PAGE separation, protein bands were labeled by silver staining. For western blot analysis see DARTS analysis.

## Results and discussion

**Fig 2:**
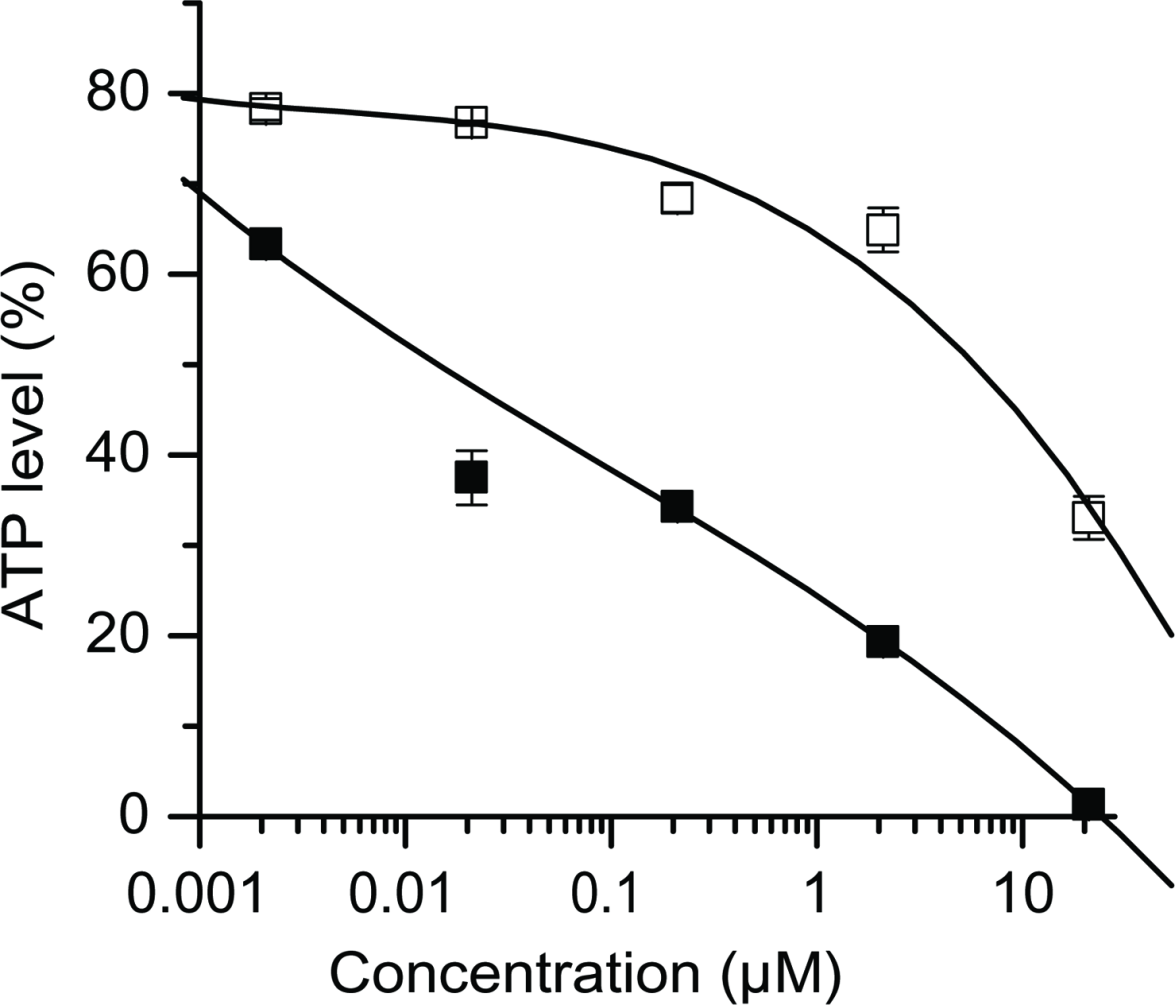
Activity of GA in primary and transformed cells. Human dermal fibroblasts (NHDF; □) and cancerous KB-3-1 cells (■) were incubated with different concentrations of GA and measured for viability after 48 hours. NHDFs were less sensitive than KB-3-1 cancer cells.

GA has been reported to effectively inhibit the proliferation of mammalian cells in culture [6]. By feeding precursors of the main metabolic pathways it has also been shown that GA inhibits protein biosynthesis. In order to confirm these results and to dissect the mode of action of GA, cellular and *in vitro* tests were performed. In additional tests with transformed cell lines, we also measured a high antiproliferative potential of GA (S1 File). For candidaspongiolide, another translation inhibitor which shows structure similarities to GA, it has been reported that normal human fibroblasts are much less sensitive than cancer cell lines [11]. We therefore tested whether GA has a similar behavior and compared the toxicity of GA in cancerous KB-3-1 cells and normal fibroblasts. The ATP content of the cells was measured as a parameter of viability (Fig 2). The toxicity curves of both cell lines run rather flat indicating a less drastic effect of GA but KB-3-1 cells were clearly more sensitive than normal cells. After 48 hours of incubation, the IC_50_ was 0.015 μM with KB-3-1 cells compared to 6 μM with NHDF. Tests, as reported with the translation inhibitors pateamine A and des-methyl-des-amino pateamine A (DMDA-pateamine A) did not show a selective effect n cancer cells [12]. Pateamines inhibit translation by interfering with eukaryotic initation factor eIF4A [13], while candidaspongiolide acts by increasing the phosphorylation of the initiation factor eIF2α. But it did not induce phosphorylation of eIF2α in primary fibroblast cells [11].

**Fig 3:**
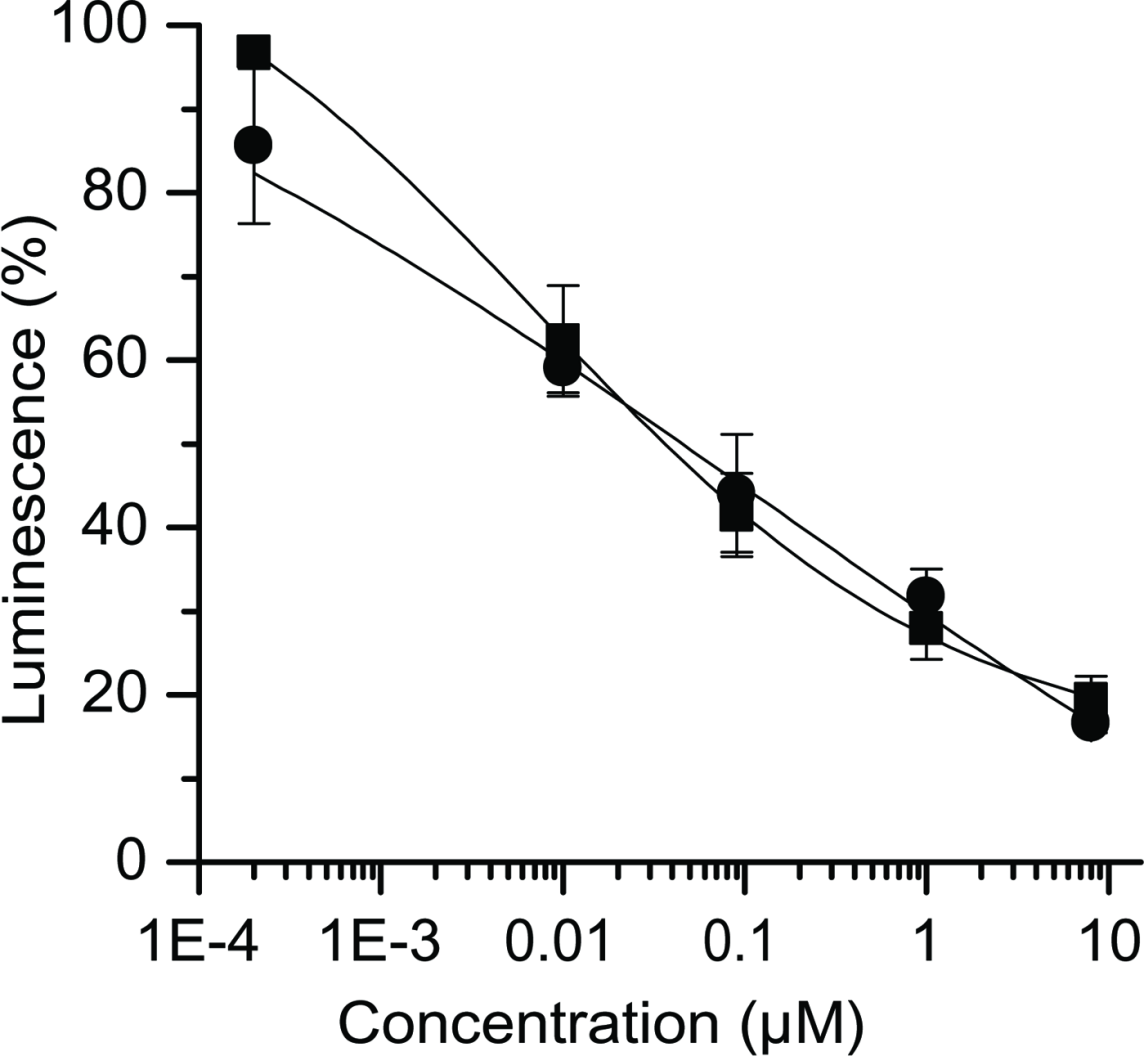
Inhibition of a luciferase signal in KB-3-1 reporter cells. GA (■) inhibits the production of *Renilla* luciferase in the cells to the same extend as cycloheximide (●). The experiments were run in triplicates. The error bars show standard deviations.

Since labeling with feeding of precursors of the main metabolic pathways proved that GA only inhibits protein synthesis [6], we could use KB-3-1 reporter cells transfected with a pRLSV40 vector containing cDNA encoding for *Renilla* luciferase to measure the cellular translation inhibition. With these cells GA exerted a strong inhibition of the luciferase signal (IC_50_ 0.04 μM) that was comparable to the effect of cycloheximide (IC_50_ 0.05 μM; Fig 3).

**Fig 4:**
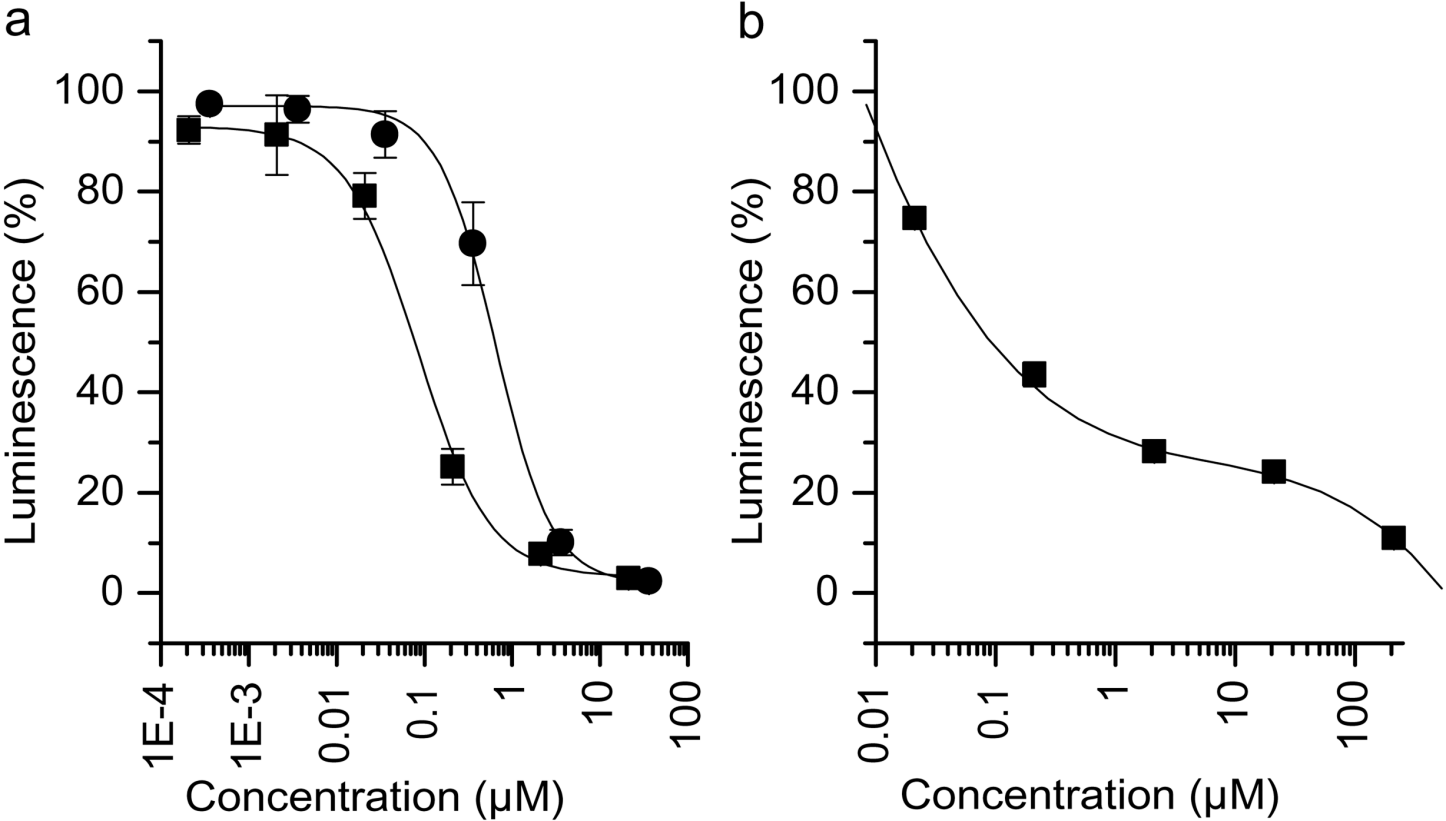
Inhibition of translation in two *in vitro* systems. (a) GA (■) inhibited the translation in a rabbit reticulocyte lysate more efficiently than cycloheximide (●). The error bars show standard deviations. (b) GA also inhibited a wheat germ lysate translation system. Here, the relative standard deviations were < 5%. The error bars do not exceed the square symbols. All experiments were run in triplicates.

A rabbit reticulocyte lysate system was used resulting in a luminescence signal as a direct measure of RNA translation. GA inhibited this *in vitro* system more efficiently than cycloheximide (IC_50_ 0.08 and 0.6 μM, resp.; Fig 4A) and was also active in wheat germ extracts (IC_50_ 0.1 μM; Fig 4B). However, GA failed to inhibit prokaryotic translation in an E. coli S30 extract (data not shown).

The results confirm that GA is a highly effective, specific inhibitor of eukaryotic protein synthesis. The translation process can be divided into at least three phases, initiation, elongation and termination. Initiation of translation usually involves the interaction of certain key proteins with a special tag, a guanine nucleotide bound to the 5'-end of the mRNA molecule, the 5' cap. An Internal Ribosome Entry Site (IRES) is a nucleotide sequence that allows for cap-independent translation initiation in the middle of the mRNA sequence. Different IRES sequences are used by several viruses to ensure that viral translation is active while host translation is inhibited [14]. These IRES sequences can be applied to elucidate the stage of translation inhibition in the cell. We used two bicistronic reporter systems in transfected KB-3-1 cells with a firefly luciferase gene that is translated by a cap-dependent translation and a *Renilla* luciferase which is translated via two different IRES sequences, either a polio IRES or a cricket paralysis virus IRES. Cap-dependent firefly luciferase translation requires the tightly regulated initiation factors for cap binding, scanning of the mRNA and assembly of the ribosome by joining of the two ribosomal subunits. However, polio IRES mediated translation does not require the initiation factor eIF4E. The 43S complex is only recruited with the help of eIF4A and eIF4G. Cricket paralysis virus (CrPV) IRES does not require the entire set of initiation factors for translation [15–18]. DMDA-pateamine A, which inhibits translation initiation by interfering with eIF4A, and cycloheximide, which inhibits the elongation phase, were used as controls [13, 19, 20].

**Fig 5:**
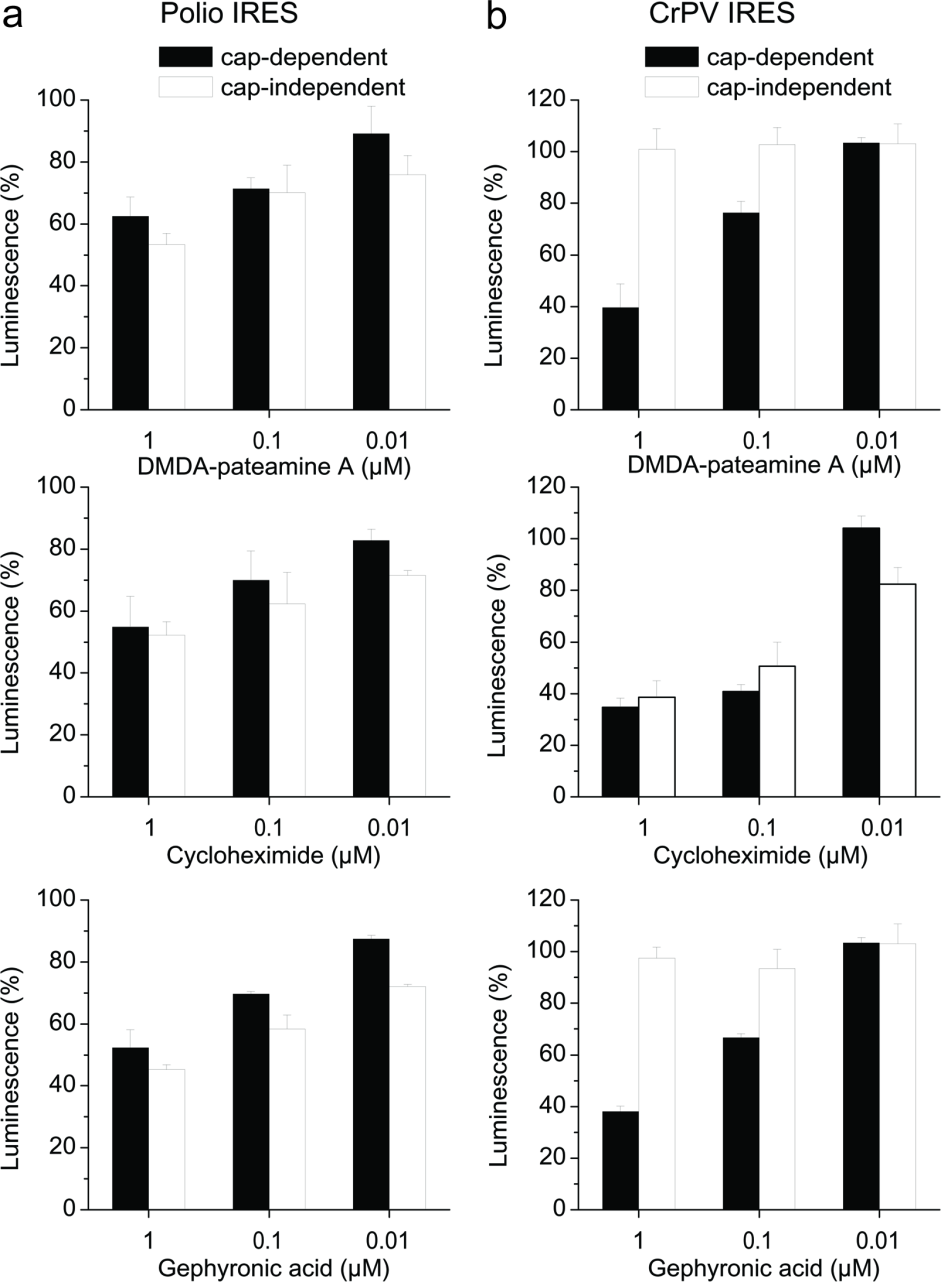
Bicistronic reporter systems in KB-3-1 cells allowing to compare cap-dependent translation directly to cap-independent in the same environment. GA inhibited the translation of the polio IRES sequence (a) but not that of the CrPV IRES (b). DMDA-pateamine A which targets eIF4A also inhibited the polio IRES and not the CrPV IRES mediated translation. Cycloheximide, which targets the elongation phase, inhibited translation from both IRES sequences. All experiments were run in triplicates. The error bars show standard deviations.

GA inhibited the cap independent translation in the polio IRES construct to the same extend as cap-dependent translation, but did not inhibit the CrPV IRES mediated translation of the *Renilla* luciferase mRNA. The result is similar to that of DMDA-pateamine A. Cycloheximide inhibited both IRES mediated translations to the same extend as the cap-dependent translation (Fig 5). The results suggest that the target of GA lies within the initiation phase of translation, but it is not eIF4E.

**Fig 6:**
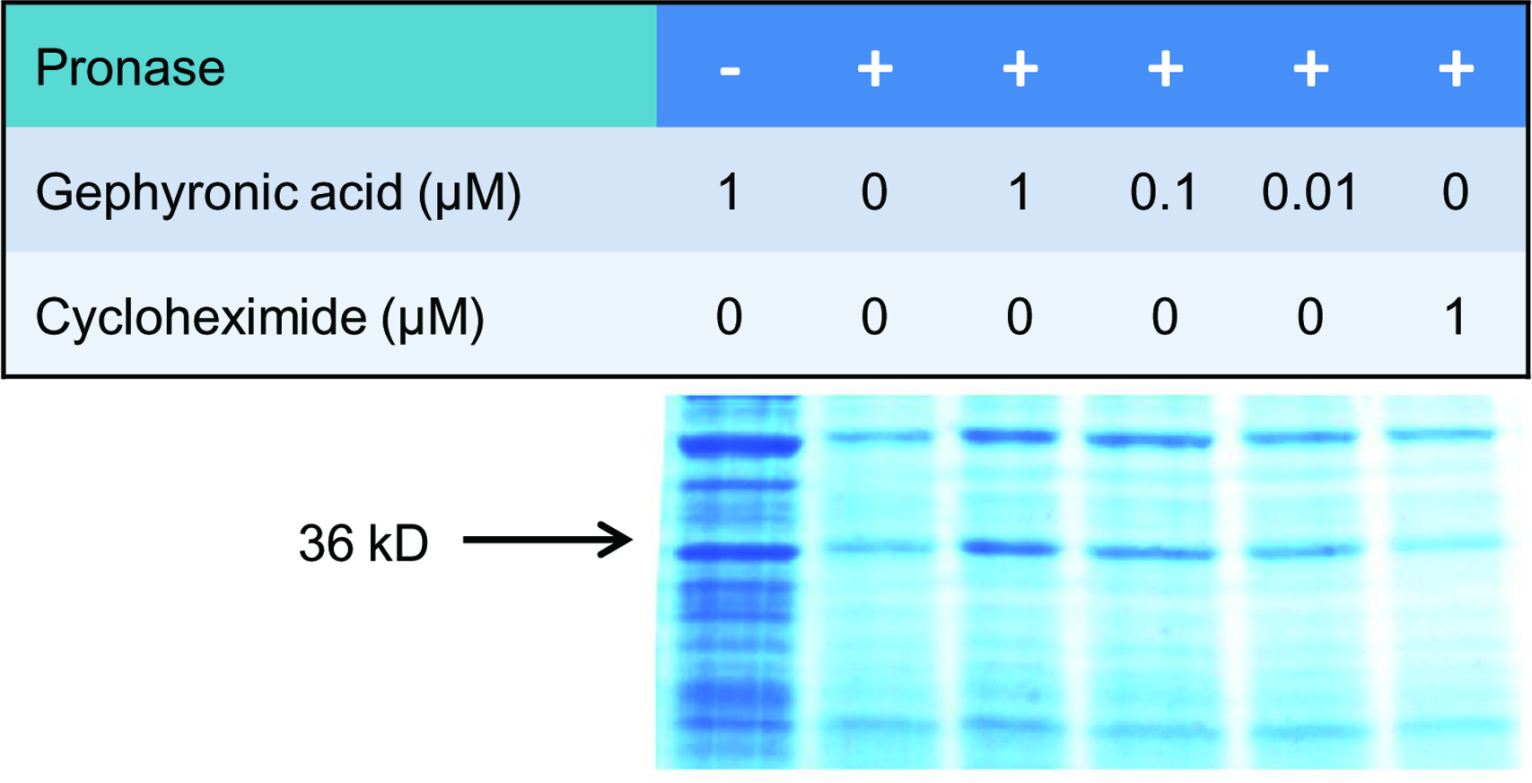
DARTS approach with GA. KB-3-1 cell lysates incubated with different concentrations of GA were subjected to pronase digestion. After SDS-PAGE protein bands were stained with Coomassie Brilliant Blue. A lysate treated with cycloheximide served as negative control. GA partially protected a protein at 36 kDa (see arrow) from digestion that is not protected by cycloheximide.

In order to identify the target protein of GA we used a drug affinity responsive target stability (DARTS) approach. The basis of this method is that binding of a ligand to a protein reduces its susceptibility to protease digestion due to a stabilization of the protein’s folded state [21]. We incubated KB-3-1 cell lysates with GA and subjected them to pronase digestion at 37°C for 30 min. After PAGE separation we observed a protein band at 36 kDa that seemed to be at least partially protected by GA. Cycloheximide, which was used as a negative control, failed to protect this band from digestion (Fig 6).

**Fig 7:**
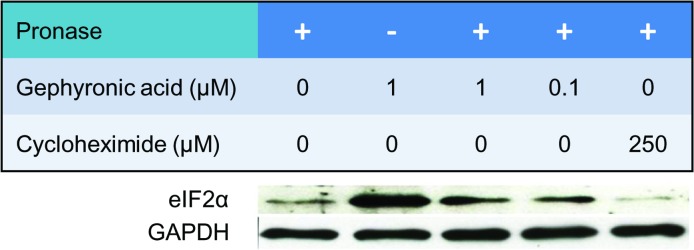
Western blot for eIF2α following a DARTS approach. KB-3-1 cell lysates treated with varying concentration of GA were subjected to DARTS analysis with pronase. Lysates treated with cycloheximide served as negative control. GA binding protected the initiation factor eIF2α in a dose dependent manner. GAPDH was used as a loading control.

A decisive factor in translation initiation having a size of 36 kDa is eIF2α. eIF2α is a subunit of eIF2, a GTP-binding protein responsible for bringing the initiator tRNA to the P-site of the pre-initiation complex. Using Western blotting a protein at 36 kDa was indeed identified as eIF2α (Fig 7). This protein was clearly protected from pronase digestion by GA in a concentration dependent manner. It was not protected by cycloheximide, even at high amounts. The DARTS experiments indicate that GA binding directly protects eIF2α from pronase degradation.

**Fig 8:**
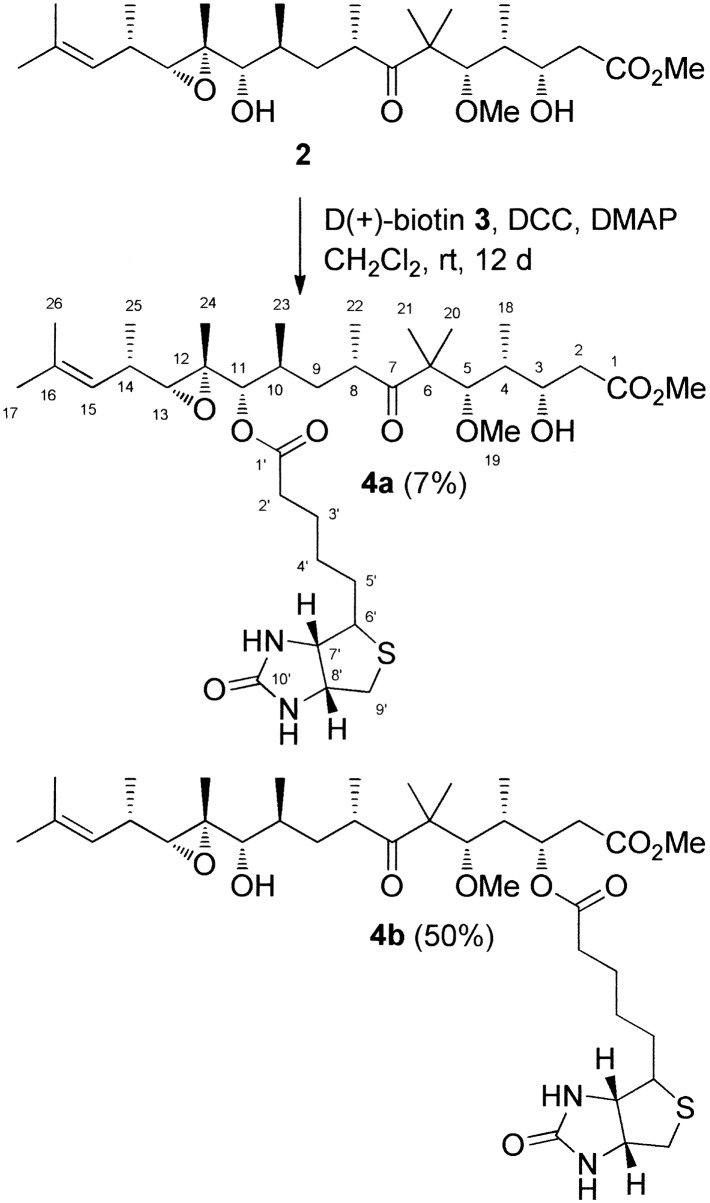
Biotinylation of GA methylester 2. Steglich esterification resulted the two biotinylated derivatives **4a** and **4b**.

In order to fish the target protein we synthesized biotinylated derivatives of GA methyl ester **2.** The methylester **2** was used instead of the acid **1**, because previous SAR studies showed a higher activity for **2** as compared to the natural product **1** in cell proliferation assays [22]. Via Steglich esterification we obtained two biotinylated products with a biotin fragment attached to either the hydroxyl group at C11 (**4a**) or at C3 (**4b**; Fig 8 and S2 File). Tests with KB-3-1 cells showed that the biotinylated derivatives were still active, but the C3 derivative **4b** was more active (IC_50_ 0.06 μM) than **4a** biotinylated at C11 (IC_50_ 0.18 μM); S3 File). These results confirm earlier findings that the epoxide is important for the activity of GA [22]. It was also shown that **4b** retained the ability to interfere with protein biosynthesis. It inhibited the translation in an *in vitro* reticulocyte system with an IC_50_ of 0.6 μM (S3 File).

**Fig 9:**
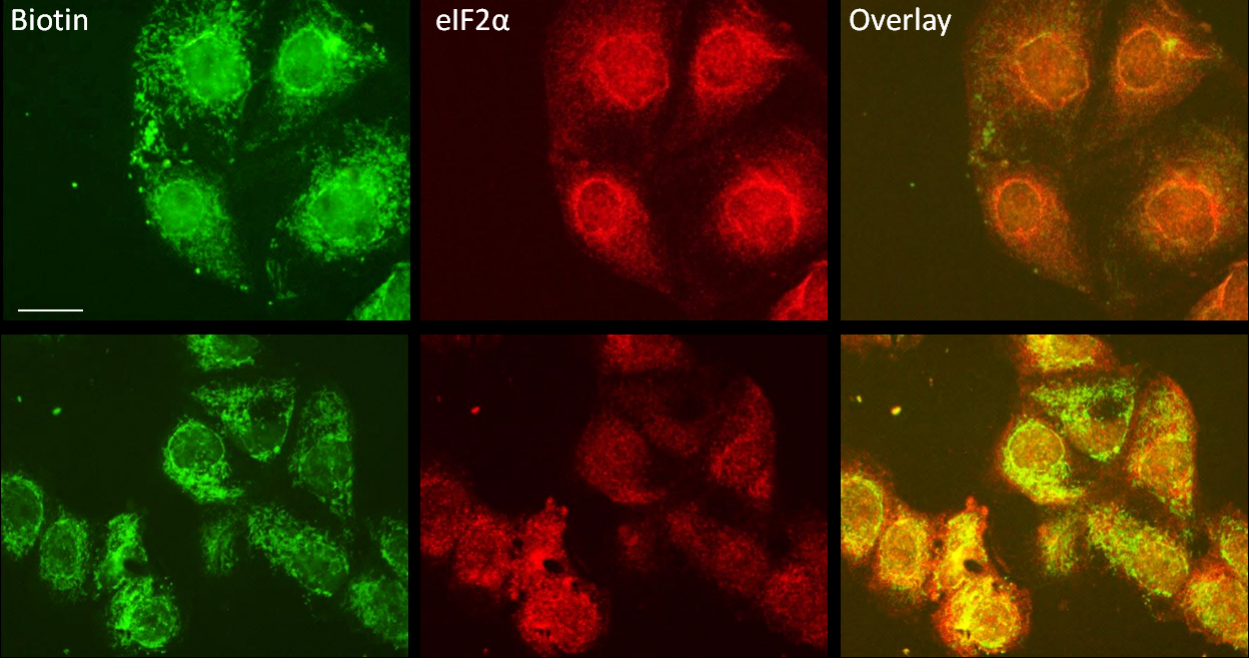
Co-localization studies with cultivated cells. PtK2 cells were treated with 50 nM of GA (upper row) or biotinylated GA methyl ester (lower row) for three hours and stained for eIF2α (red) and biotin (green). The green biotin label often co-localizes with the red one of eIF2α shown by the resulting yellow color in the overlay image (bottom left).

Protein synthesis in cells is taking place at ribosomes which are not always homogenously distributed in the cell, e.g., they show changing distribution patterns in dependence of the cell cycle phase [23]. We therefore tried to use the biotin GA methyl ester **4b** to show the putative co-localization with eIF2α in the ribosomes of a cultured cell population. Transformed PtK2 cells from *Potorous tridactylis* were used for fluorescence staining analyses, because these cells are flat and more appropriate for microscopic studies. Cells were incubated with **4b** and then stained for biotin by adding green labeled streptavidin, and for eIF2α by using specific primary and red labeled secondary antibodies. Overlaying of the two labels resulted in a yellow color, thereby showing a co-localization of the GA derivative and eIF2α (Fig 9). Additional green biotin label is probably due to natural biotin in the cell.

**Fig 10:**
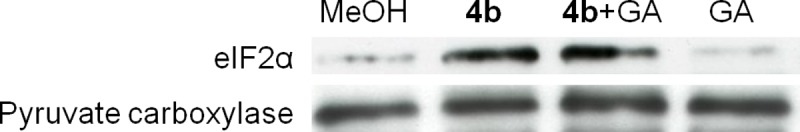
Pull-down assay. KB-3-1 cell lysates were incubated with methanol, GA or biotinylated GA methyl ester and the targets pulled down using a streptavidin column. The eluates were analyzed by western blotting. The samples from the lysates treated with C3-biotinylated GA methyl ester showed clear bands of eIF2α at 36 kDa that were almost not present in the preparations with methanol or GA only. Biotin containing pyruvate carboxylase, which is also fished, served as a control.

In pull-down assays KB-3-1 cell lysates were incubated with the biotinylated GA ester **4b,** and bound proteins were fished with streptavidin coated beads. Proteins were eluted under denaturing conditions and separated by PAGE. The samples treated with the biotinylated GA ester showed a faint band at a molecular mass of 36 kDa, which was less pronounced in the control lysates. Using western blotting we again identified eIF2α (Fig 10). eIF2α was clearly present in preparations with biotinylated GA ester but almost absent in samples with methanol and GA only. The blot was also probed with an antibody against pyruvate carboxylase. Pyruvate carboxylase is a biotin containing protein present in the mitochondria which also binds to streptavidin and is also fished. So pyruvate carboxylase served as a positive control for the pull-down assay.

The DARTS approach showed a 36 kDA protein that seemed to be protected by GA. Western blotting identified the initiation factor eIF2α. The amounts were depending on the GA concentration. Pull-down experiments also fished a 36 kDa protein that was again identified as eIF2α. Cell staining experiments showed a co-localization of biotinylated GA ester and eIF2α. The combined results of these experiments lead to the hypothesis that the primary target of GA could be eIF2α. eIF2α is part of the trimeric complex eIF2. The α-subunit contains the main target site for phosphorylation, and also contains an S1 motif domain, which is a potential RNA binding-site. Therefore, the α-subunit can be considered the regulatory subunit of the trimer.

*In silico* docking studies resulted in a binding suggestion that was also compatible with microarray-based peptide interaction analysis (S4 and S5 Files). In order to check our binding hypothesis *in vitro* we produced heterologous eIF2α protein in High Five insect cells by transient gene expression (TGE) and tested it for binding to biotinylated GA methyl ester **4b** by pull-down experiments (S6 File). The results did not show a binding to the heterologously expressed proteins. Although we were not able to proof our hypothesis *in vitro*, we have to consider that our *in vitro* conditions do not necessarily provide the decisive binding conformation present *in vivo*, where eIF2α is only one subunit of the heterotrimeric complex eIF2.

While enrichment of eIF2α in DARTS and pull down experiments has been convincingly shown, it should be emphasized that upregulation/stabilization due to protein modification, protein complex modulation or upregulation of upstream targets, although not very likely, cannot be completely ruled out.

Notwithstanding, GA clearly inhibits the initiation of the eukaryotic protein synthesis. This was shown by two bicistronic reporter experiments (Fig 5). The results are compatible with the hypothesis that the target of GA could be eIF2α. The polio IRES mediated translation, which needs eIF2α, is inhibited while the CrPV IRES controlled translation, which is not depending on eIF2α, is not inhibited. eIF2α is highly conserved in eukaryotes, but differs greatly from its prokaryotic counterpart. Gephyronic acid showed no activity against prokaryotes and did not inhibit prokaryotic *in vitro* translation.

GA has a completely different mode of action compared to the structurally related translation inhibitor myriaporone 3/4, which also has a polyketide backbone but shows differences in the arrangements of hydroxyl groups which are possible functional elements for the interaction with target proteins. Bicistronic reporter assays showed that myriaporone 3/4 inhibits the elongation of translation. DARTS results suggest that it possibly binds to eEF2 kinase [24]. Desepoxy-tedanolide was shown to induce a phosphorylation of eEF2 and of eIF2α, but the primary target is not yet known [25, 26].

How could binding of GA to eIF2α stall the translation process? The eIF2 activity is regulated by a mechanism involving both guanine nucleotide exchange and phosphorylation. Phosphorylation takes place at the α-subunit. In humans, this is exerted by four different eIF2α kinases [27]. Once phosphorylated, eIF2 shows increased affinity for its guanine nucleotide exchange factor (GEF) eIF2B. However, eIF2B is able to exchange GDP for GTP only if eIF2 is in its unphosphorylated state. Phosphorylated eIF2 acts as an inhibitor of its own GEF (eIF2B). Since the cellular concentration of eIF2B is much lower than that of eIF2, even a small amount of phosphorylated eIF2 can completely sequester eIF2B activity. Without the GEF, GDP cannot be exchanged for GTP, and eIF2 not be returned to its active (GTP-bound) state. Preliminary results of immunofluorescence staining of cells incubated with GA showed an enhanced phosphorylation of eIF2α. This would limit the availability of eIF2∙GTP∙Met-tRNA_i_^Met^ ternary complexes (TC) and reduce the formation of 43S pre-initiation complexes. NSC119889, a published translation inhibitor, blocks the TC formation thereby suppressing cap-dependent translation but not the translation driven by HCV IRES [28], and moreover the reduced availability of TC stimulated the translation mediated by the CrPV IRES [29]. This was not observed in our experiments with CrPV IRES (Fig 5). However, experiments with heterologously expressed eIF2α protein did not show a direct binding to GA. We therefore do not have a direct physical proof for the binding site of GA and the docking studies have to be considered as a working hypothesis. Further experiments, *e*.*g*., with GA resistant cell lines, have to be undertaken to finally find out how GA is acting on the complex eukaryotic protein translation system.

## Conclusions

GA is a nM inhibitor of the eukarotic protein translation process. Cancer cells seem to be more sensitive than normal healthy fibroblasts. Bicistronic reporter assays showed that GA inhibits protein translation in its initiation phase. Results from DARTS and pull-down experiments with incubated cells suggest that eIF2α could be the primary target protein. Interestingly all the structurally related members of the polyketide family consisting of tedanolide, myriaporone, gephyronic acid, and pederin are translation inhibitors but each inhibitor seems to act in a different way. The direct binding partners are not known so far. The binding of GA with heterologously expressed eIF2α could not be shown, possibly because the complex *in vivo* conditions cannot easily be reproduced *in vitro*. Although the direct proof is still missing, our results suggest that eIF2α is the target protein of GA. Genetic engineering of eIF2α mutants as well as screening and sequencing of GA resistant cells lines could be feasible ways to pinpoint the GA target in future studies.

## Supporting information

S1 File Growth inhibition of gephyronic acid with mammalian cell lines. (PDF)

S2 File NMR analyses of biotin esters. (PDF)

S3 File Biological activity of biotinylated gephyronic acid methyl ester. (PDF)

S4 File Docking studies. (PDF)

S5 File Peptide mapping. (PDF)

S6 File Binding assays with heterologous eIF2α protein. (PDF)
