# Supplementary material for: Investigations on the mode of action of gephyronic acid, an inhibitor of eukaryotic protein translation from myxobacteria
Source: PLoS One. 2018 Jul 31;13(7):e0201605. doi: 10.1371/journal.pone.0201605 (PMC6067752; doi:10.1371/journal.pone.0201605)
Supplement: S1 File — (PDF) [file pone.0201605.s001.pdf]

S1 File. Growth inhibition of gephyronic acid with mammalian cell lines

Anti-proliferative activity of gephyronic acid was measured in 96-well plates. 60 µl of a serial dilution of myriaporone were added to 120 µl of suspended cells (50,000 cells/ml; two replicates). The end concentrations tested ranged from 37 µg to 2 ng/ml. After five days the metabolic activity in each well was determined using MTT (3-(4,5-dimethylthiazol-2-yl)-2,5-diphenyltetrazolium bromide). 20 µl MTT in PBS were added to a final concentration of 0.5 mg/ml and incubated for 2 h. MTT is reduced then by dehydrogenases of the cells to form purple formazan crystals. The precipitate was washed with 100 µl PBS and dissolved in 100 µl isopropanol containing 0.4 % hydrochloric acid. The resulting color was measured at 595 nm using a plate reader. The IC_{50} was calculated in relation to control cells that were treated with the vehicle only.

Table: Anti-proliferative activity of gephyronic acid with different transformed cell lines

Cell Line[a]	Origin	Gephyronic acid IC_{50} [µM][b]
L-929	Mouse connective tissue	0.10
KB-3-1	Human cervical cancer	0.014
PC-3	Human prostate cancer	0.006
A-549	Human lung cancer	0.008
MCF-7	Human breast cancer	0.014

[a] Cells were obtained from DSMZ or ATCC; [b] IC_{50} values are means of two assays in parallel. The relative standard deviations do not exceed 20%.
