# Supplementary material for: Investigations on the mode of action of gephyronic acid, an inhibitor of eukaryotic protein translation from myxobacteria
Source: PLoS One. 2018 Jul 31;13(7):e0201605. doi: 10.1371/journal.pone.0201605 (PMC6067752; doi:10.1371/journal.pone.0201605)
Supplement: S2 File — (PDF) [file pone.0201605.s002.pdf]

S2 File. NMR analyses of biotin esters

(C-11)-biotin ester (**4a**) = Methyl (3*S*,4*R*,5*S*,8*S*,10*S*,11*S*)-11-hydroxy-5-methoxy-4,6,6,8,10-pentamethyl-11-{(2*R*,3*R*)-2-methyl-3-[(*S*)-4-methylpent-3-en-2-yl]oxiran-2-yl}-7-oxo-11-{5-{(3a*S*,6a*R*)-2-oxohexahydro-1*H*-thieno[3,4-d]imidazol-4-yl}pentanoyloxy}un-decanoate

R_f = 0.35 (ethyl acetate/MeOH 10 : 1). ^{1}H-NMR (800 MHz, $CDCl_3$) δ = 0.90 (d, *J* = 6.3 Hz, 3H, 23-H), 0.92 (d, *J* = 7.0 Hz, 3H, 18-H), 1.00 (d, *J* = 6.3 Hz, 3H, 22-H), 1.06-1.16 (m, 1H, 9-H_b), 1.095 (d, *J* = 6.0 Hz, 3H, 25-H), 1.101 (s, 3H, 21-H), 1.21 (s, 3H, 20-H), 1.28-1.37 (m, 1H, 9-H_a), 1.39 (s, 3H, 24-H), 1.47-1.50 (m, 1H, 4'-H_b), 1.50-1.57 (m, 1H, 4'-H_a), 1.60-1.69 (m, 3H, 4-H, 5'-H_b, 3'-H_b), 1.69 (s, 3H, 26-H), 1.71 (s, 3H, 17-H), 1.73-1.80 (m, 3H, 10-H, 5'-H_a, 3'-H_a), 2.35-2.40 (m, 1H, 2'-H_b), 2.44-2.47 (m, 1H, 2'-H_a), 2.47-2.50 (m, 1H, 2-H_b), 2.52-2.60 (m, 2H, 2-H_a, 14-H), 2.71-2.75 (m, 2H, 9'-H_b, 13-H), 2.92-2.97 (m, 1H, 9'-H_a), 3.05-3.10 (m, 1H, 8-H), 3.16-3.21(m, 1H, 6'-H), 3.36 (2, 3H, 19-H), 3.52-3.54 (m, 1H, 5-H), 3.73 (s, 3H, COO*Me*), 3.94-3.99 (m, 1H, 3-H), 4.25-4.29 (m, 1H, 11-H), 4.35-4.40 (m, 1H, 7'-H), 4.51-4.55 (m, 1H, 8'-H), 4.72 (brs, 1H, N*H*), 4.94-4.99 (m, 1H, 15-H), 5.66 (brs, 1H, N*H*) ppm. ^{13}C-NMR (200 MHz, $CDCl_3$) δ = 8.8 (C-18), 12.2 (C-24), 15.1 (C-23), 15.9 (C-22), 18.3 (C-26), 19.1 (C-21), 20.5 (C-20), 21.2 (C-25), 25.1 (C-3'), 25.8 (C-17), 28.2 (C-5'), 28.4 (C-4'), 32.2 (C-14), 32.4 (C-10), 34.3 (C-2'), 36.6 (C-9), 37.7 (C-8), 39.3 (C-2), 39.7 (C-4), 40.6 (C-9'), 51.9 (COOMe), 52.0 (C-6), 55.6 (C-6'), 60.1 (C-8'), 60.3 (C-19), 61.6 (C-7'), 62.7 (C-12), 68.6 (C-13), 70.8 (C-3), 82.7 (C-11), 86.3 (C-5),124.8 (C-15), 132.5 (C-16), 163.0 (C-10'), 172.9 (C-1'), 173.4 (C-1) ppm. MS (ESI) *m/z* = 733 $[M+Na]^+$, 693, 449. HRMS (ESI) *m/z*: calcd. for $C_{37}H_{62}N_2O_9SNa^+$ = 733.4068, found: 733.4076 $[M+Na]^+$. FT-IR (ATR) $\tilde{\nu}$ = 3283 (m), 2924 (vs), 2854 (s), 2671 (w), 2483 (w), 2359 (w), 1735 (s), 1697 (s), 1558 (w), 1460 (s), 1378 (m), 1256 (m), 1164 (m), 1099 (m), 989 (m) cm^{-1}. $[\alpha]_D^{20}$ = + 2.6 (c = 1.00, CH_2Cl_2)

(C-3)-biotin ester (**4b**) = Methyl (3*S*,4*R*,5*S*,8*S*,10*S*,11*S*)-11-hydroxy-5-methoxy-4,6,6,8,10-pentamethyl-11-{(2*R*,3*R*)-2-methyl-3-[(*S*)-4-methylpent-3-en-2-yl]oxiran-2-yl}-7-oxo-3-{5-{(3a*S*,6a*R*)-2-oxohexahydro-1*H*-thieno[3,4-d]imidazol-4-yl}pentanoyloxy}un-decanoate

R_f = 0.30 (ethyl acetate/MeOH 10 : 1). ^{1}H-NMR (800 MHz, $CDCl_3$) δ = 0.81 (d, *J* = 6.7 Hz, 3H, 23-H), 0.96 (d, *J* = 7.0 Hz, 3H, 18-H), 1.02 (d, *J* = 6.5 Hz, 3H, 22-H), 1.10 (s, 3H, 21-H), 1.11 (d, J = 6.7 Hz, 3H, 25-H), 1.19-1.24 (m, 1H, 9-H_b), 1.23 (s, 3H, 20-H), 1.27 (s, 3H, 24-H), 1.43-1.56 (m, 3H, 4'-H_b, 4'-H_a, 10-H), 1.61-1.71 (m, 2H, 5'-H_b, 3'-H_b), 1.64 (s, 3H, 26-H), 1.70 (s, 3H, 17-H), 1.72-1.80 (m, 2H, 5'-H_a, 3'-H_a), 1.95-2.00 (m, 1H, 4-H), 2.31-2.35 (m, 1H, 14-H), 2.35-2.38 (m, 1H, 2'-H_b), 2.43-2.48 (m, 1H, 2'-H_a), 2.60-2.66 (m, 3H, 13-H, 2-H_b, 2-H_a), 2.71-2.78 (m, 2H, 11-H, 9'-H_b), 2.92-2.97 (m, 1H, 9'-H_a), 3.13-3.22 (m, 2H, 8-H, 6'-H), 3.31 (2, 3H, 19-H), 3.46-3.48 (m, 1H, 5-H), 3.70 (s, 3H, COO*Me*), 4.34-4.38 (m, 1H, 7'-H), 4.52-4.56 (m, 1H, 8'-H), 4.95-4.99 (m, 1H, 15-H), 5.17-5.21 (m, 1H, 3-H), 5.27 (brs, 1H, N*H*), 5.57 (brs, 1H, N*H*) ppm. ^{13}C-NMR (200 MHz, $CDCl_3$) δ = 9.9 (C-18), 11.4 (C-24), 15.7 (C-23), 16.4 (C-22), 18.4 (C-26), 21.7 (C-21), 19.0 (C-25), 19.8 (C-20), 24.8 (C-3'), 25.9 (C-17), 28.19 (C-5'), 28.21 (C-4'), 32.5 (C-14), 33.4 (C-10), 34.1 (C-2'), 36.9 (C-2), 37.5 (C-9), 37.6 (C-4), 38.3 (C-8), 40.6 (C-9'), 51.9 (COOMe), 54.0 (C-6), 55.2 (C-6'), 60.2 (C-8'), 60.4 (C-19), 61.9 (C-7'), 64.4 (C-12), 68.4 (C-13), 73.6 (C-3), 82.7 (C-11), 84.9 (C-5),125.0 (C-15), 132.1 (C-16), 163.5 (C-10'), 171.0 (C-1), 172.9 (C-1') ppm. MS (ESI) *m/z* = 733 $[M+Na]^+$, 711, 693, 599. HRMS (ESI) *m/z*: calcd. for $C_{37}H_{62}N_2O_9SNa^+$ = 733.4068, found: 733.4067 $[M+Na]^+$. FT-IR (ATR) $\tilde{\nu}$ = 3260 (m), 2924 (vs), 2854 (s), 2558 (w), 2365 (w), 2183 (w), 1967 (w), 1736 (s), 1698 (s), 1456 (s), 1377 (m), 1260 (s), 1160 (m), 1099 (s), 989 (m), 801 (m) cm^{-1}. $[\alpha]_D^{20}$ = + 2.0 (c = 1.00, CH_2Cl_2)

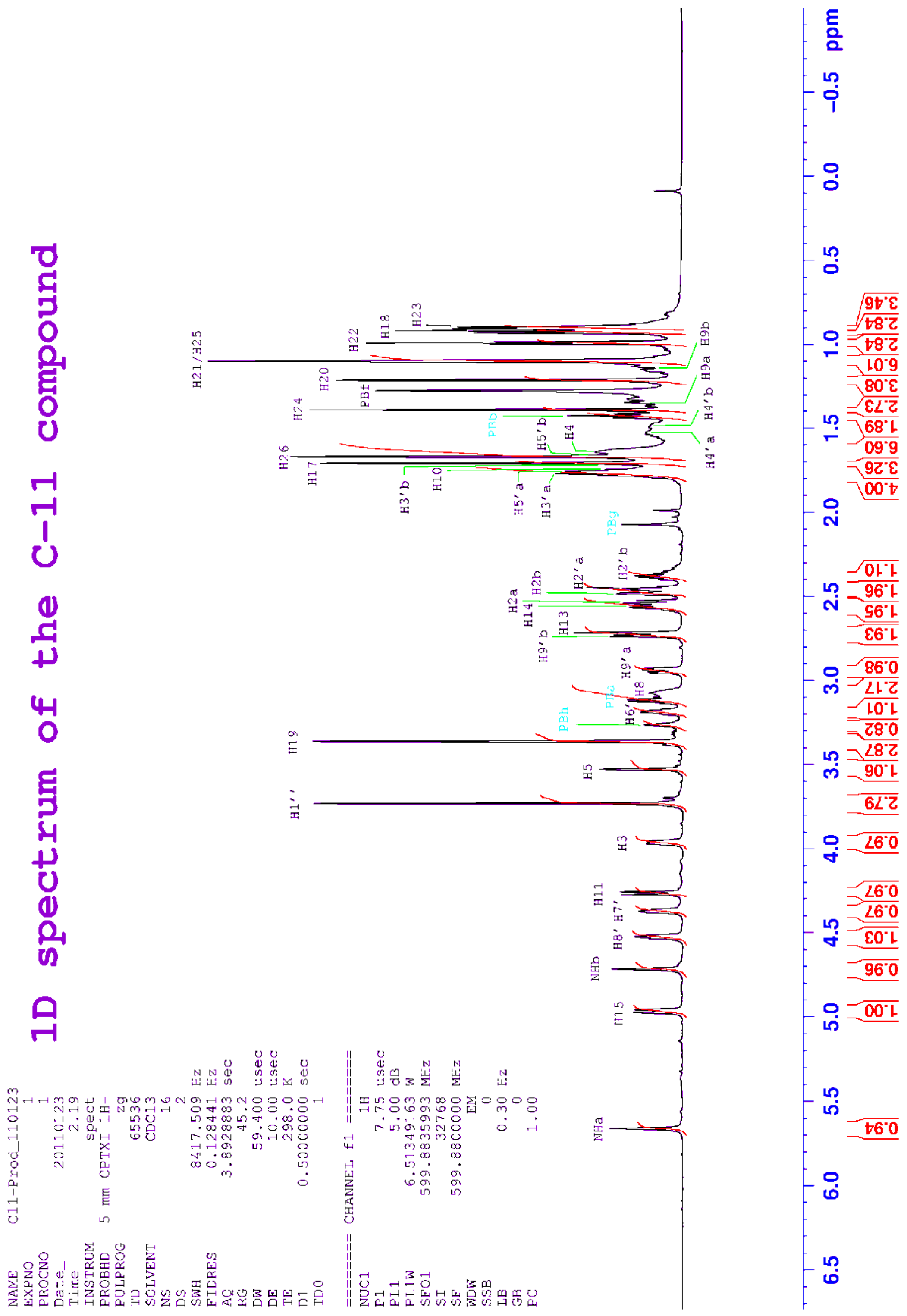

^{1}H-NMR spectrum (600 MHz, $CDCl_3$) of (C-11)-biotin ester 4a

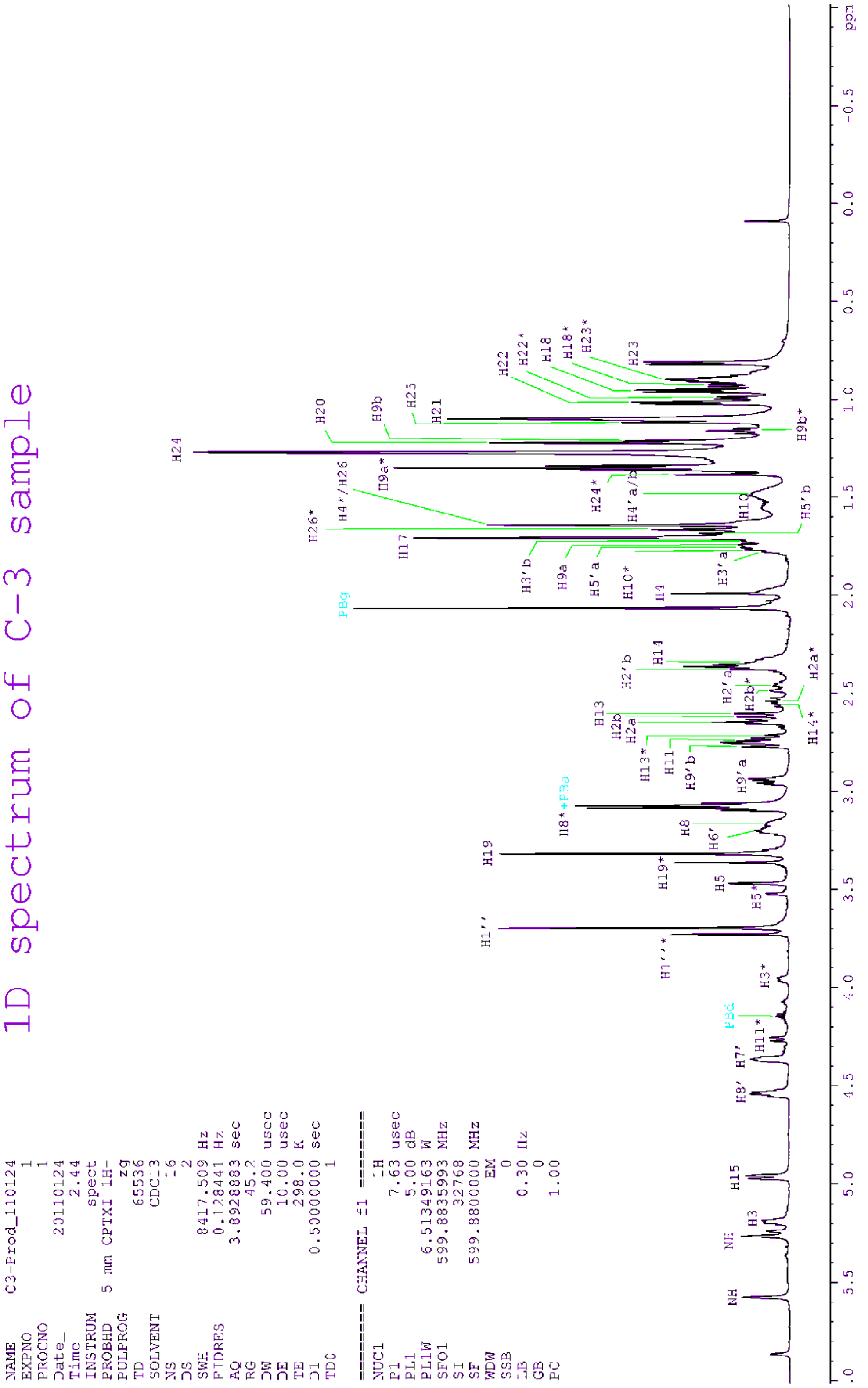

^{1}H-NMR spectrum (600 MHz, $CDCl_3$) of (C-3)-biotin ester 4b
