# Supplementary material for: Investigations on the mode of action of gephyronic acid, an inhibitor of eukaryotic protein translation from myxobacteria
Source: PLoS One. 2018 Jul 31;13(7):e0201605. doi: 10.1371/journal.pone.0201605 (PMC6067752; doi:10.1371/journal.pone.0201605)
Supplement: S3 File — (PDF) [file pone.0201605.s003.pdf]

S3 File. Biological activity of biotinylated gephyronic acid methyl ester

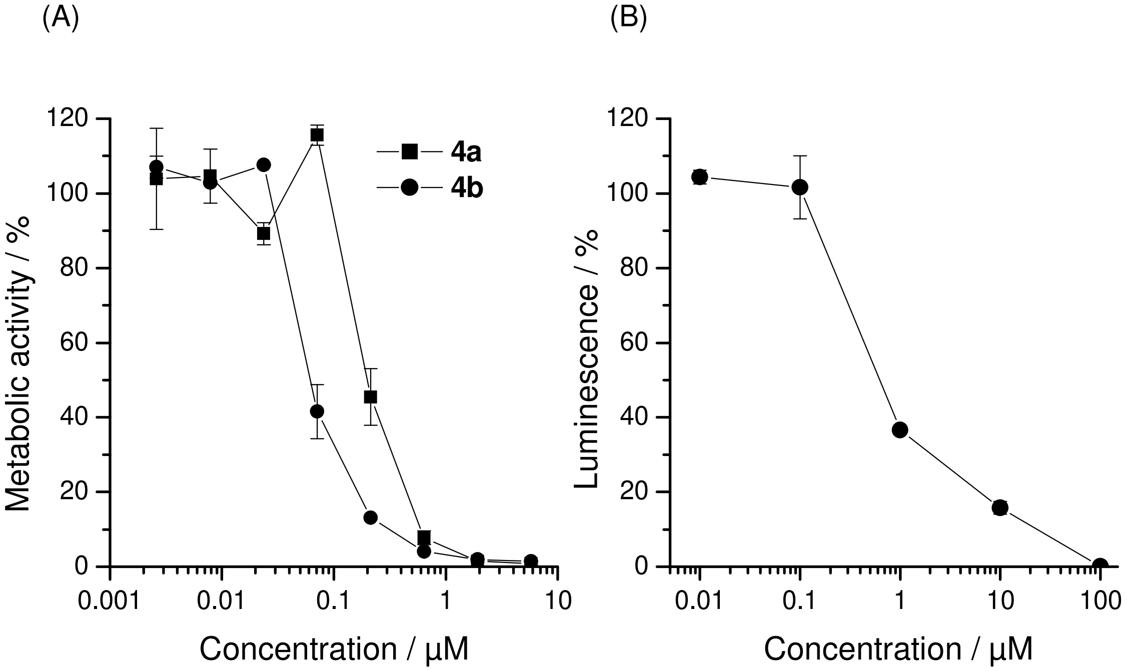

Figure: Growth and translation inhition of biotinylated gephyronic acid methyl esters. (A) Measuring the concentration dependent metabolic activity of KB-3-1 cell cultures that were incubated with **4a** and **4b** by an MTT assay showed that the compounds still had a high inhibiting capacity. **4b** was the more active one. It was almost as cytotoxic to KB-3-1 cells as the parent compound **1.** The IC_{50} was 0.06 µM. Assays were run in duplicates. Error bars show S.D. (B) **4b** also inhibited *in vitro* translation (IC_{50} 6 µM) in a rabbit reticulocyte lysate system supplemented with mRNA and amino acids. Experiment was run in triplicates. Error bars show S.D.
