# Supplementary material for: Investigations on the mode of action of gephyronic acid, an inhibitor of eukaryotic protein translation from myxobacteria
Source: PLoS One. 2018 Jul 31;13(7):e0201605. doi: 10.1371/journal.pone.0201605 (PMC6067752; doi:10.1371/journal.pone.0201605)
Supplement: S4 File — (PDF) [file pone.0201605.s004.pdf]

S4 File. Docking studies

To locate the possible binding pocket in eIF2α we performed docking calculations for the GA methyl ester **2** using Vina [1]. We used the coordinates of the target region for eIF2α specific kinases from the Protein Data Bank in Europe (PDBe), accession number 1q46, for our calculations [2], and the PRODRG server to get the coordinates and charges. The known 3D chiral centers were checked. During a first run we determined the residues involved in the interaction. In a second run we allowed torsion angle changes in all related side chains. The calculations were performed using the known 3D structure of GA.

(A)

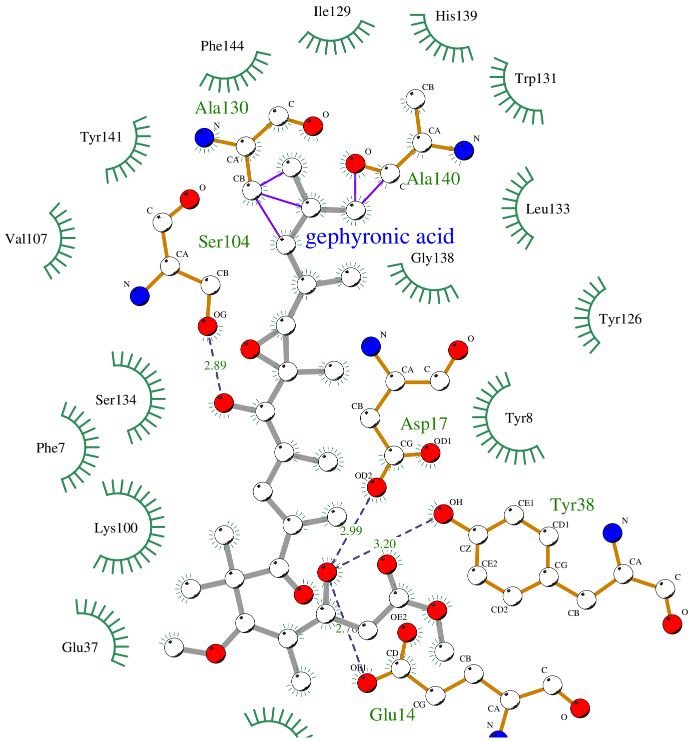

(B)

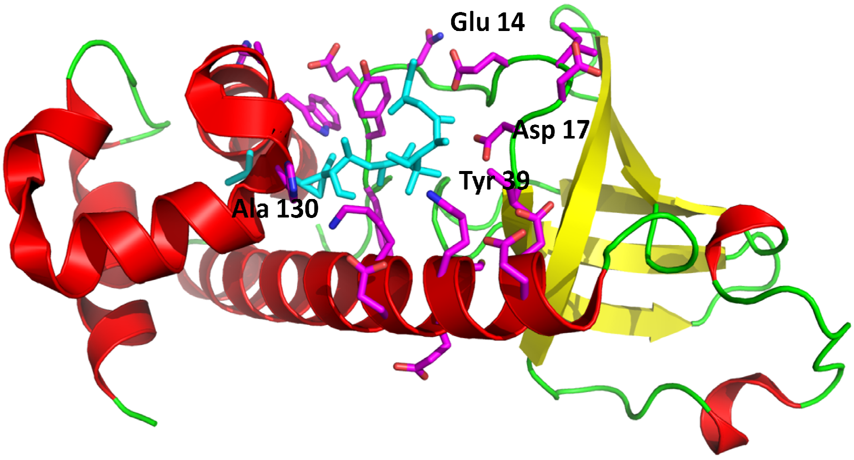

Figure: The calculated binding pocket of GA methyl ester 2 in eIF2α. (A) 2D interaction plot. The results suggest that the aliphatic ends of **2** are bound to Ala 130 and Ala 140, the carboxylic ends to Glu 14, Asp 17, and Tyr 38. (B) 3D representation of the docking solution.

References:

1. Schüttelkopf AW, van Aalten DM. PRODRG: a tool for high-throughput crystallography of protein-ligand complexes. Acta cryst 2004; D60: 1355-1363. DOI: 10.1107/S0907444904011679 PMID: 15272157

2. Dhaliwal S, Hoffman DW. The crystal structure of the N-terminal region of the alpha subunit of translation initiation factor 2 (eIF2alpha) from Saccharomyces cerevisiae provides a view of the loop containing serine 51, the target of the eIF2alpha-specific kinases. J Mol Biol 2003; 334: 187-195. PMID: 14607111
