# Supplementary material for: Investigations on the mode of action of gephyronic acid, an inhibitor of eukaryotic protein translation from myxobacteria
Source: PLoS One. 2018 Jul 31;13(7):e0201605. doi: 10.1371/journal.pone.0201605 (PMC6067752; doi:10.1371/journal.pone.0201605)
Supplement: S5 File — (PDF) [file pone.0201605.s005.pdf]

S5 File. Peptide mapping

We tried a microarray-based peptide interaction analysis, where randomly generated peptide sequences were screened for interactions with GA. Random peptide library (RPL) chips were assayed according to published protocol [1] with the difference that C3-biotinylated GA methyl ester was used instead of the first antibody and a 10% solution of a casein based blocking buffer (Genosys, 10x blocking buffer concentrate) instead of BSA. As detection system we used Cy5-labelled streptavidin. C3-biotinylated GA methyl ester (1 mg/mL) was applied at dilutions of 1:150 and 1:300 in blocking buffer and the Cy5-labelled streptavidin at 1:250 dilutions of 1 mg/mL. The developed RPL chips were scanned with an Agilent high resolution microarray scanner. Incubations with GA and Cy5-labelled streptavidin alone were used as background controls. In none of these controls any kind of binding was observed throughout the random peptides. The four most active peptide sequences showed similarities to the part of the eIF2α sequence that GA seems to interact with according to the docking calculations.

```
Q36B7   ----------------GPKYIEWNRVDCGIT-----------------------
Q32C8   --------------VLKEPQEAWMAHSRY-------------------------
Q31A12  ----------------RSWYRKQTINNMNIT-----------------------
1Q46    LEELYKTIAWPLSRKFGHAYEAFKLSIIDETVWEGIEPPSKDVLDELKNYISKR
Q20G2   -------------EMVREVCKHCGLFAM--------------------------
        ......130.......140.......150.......160.......170.....
```

Figure: Random peptide interaction analysis. The four peptides with the highest interaction showed similar amino acid sequences as the proposed binding region of 1q46.

Reference:

1. Dikmans A, Beutling U, Schmeisser E, Thiele S, Frank R. SC²: A novel process for manufacturing multipurpose high-density chemical microarrays. QSAR Comb. Sci. 2006, 25, 1069-1080. DOI:10.1002/qsar.200640130
