# Supplementary material for: Investigations on the mode of action of gephyronic acid, an inhibitor of eukaryotic protein translation from myxobacteria
Source: PLoS One. 2018 Jul 31;13(7):e0201605. doi: 10.1371/journal.pone.0201605 (PMC6067752; doi:10.1371/journal.pone.0201605)
Supplement: S6 File — (PDF) [file pone.0201605.s006.pdf]

S6 File. Binding assays with heterlogous eIF2α protein

Cloning of eIF2α

An eIF2α cDNA clone of the Mammalian Gene Collection (MGC) (GenBank BC002513) was obtained from BioCat GmbH (Heidelberg, Germany). The coding sequences of the full length protein (aa 1-303) and of the N-terminal domain (aa 1-183) were cloned between the BamHI and AvrII restriction sites of the vector pOpIE2-eGFP [1] with a C-terminal tag using the QuickFusion kit (Absource Diagnostics GmbH, Munich, Germany). The tag consists of a PreScission protease cleavage site, MBP and a 10xHis tag. The full-length and N-terminal eIF2α sequences were PCR amplified with the primer pairs eIF2a-F/eIF2a-R and eIF2a-F/eIF2a183-R (S2 Table) using the Phusion Flash High Fidelity PCR Master Mix (Thermo Fisher Scientific, Braunschweig, Germany). The tag coding sequence was amplified using primers 3CMBP-F and MBPHis-R (Tab S2) from pOPINM (GenBank EF372396). The eIF2α PCR product, the PCR product for the tag and the linearized vector were assembled by QuickFusion, followed by transformation of OmniMAX 2-T1 *E.coli* cells (Thermo Fisher Scientific).

eIF2α protein production

Full length eIF2α and the N-terminal domain were produced by transient transfection of High Five insect cells with the pOpiE2 plasmids, followed by purification of two steps of affinity chromatography. 60 ml High Five insect cells from *Trichoplusia ni* were transfected using linear 25 K polyethylenimine (PEI) as described [2], resulting in about 1 ml cell pellet. The cells were resuspended in lysis buffer (50 mM $NaHPO_4$, 0.3 M NaCl, 5 mM Imidazol, 0.5% (v/v) IGEPAL CA-630) and lysed with a Dounce homogenizer. Following centrifugation for 1 h at 50,000 rpm in a Beckman Ti-70 rotor and filtration through a 0.45 µm filter, the protein was purified by nickel chromatography with a 5 ml HisTrap column (GE Healthcare) using a wash buffer with 50 mM $NaHPO_4$ and 0.3 M NaCl (pH 8.0). For elution, 500 mM imidazol (pH 8.0) in wash buffer was used. The eluate was further purified on a MBPtrap column (GE Healthcare) using wash buffer with 20 mM HEPES and 100 mM NaCl (pH 7.4) and by elution with wash buffer containing 10 mM maltose.

Table. Primers for PCR amplifitation of eIF2α sequences

eIF2a-F	CATTTTATTTACAATCAAAGGAGATATACCATGCCGGGTCTAAGTTGTAGATTTTATCAAC
eIF2a-R	TTGCTACCCTGAAACAGAACTTCCAGATCTCCATCCACTTCGGCATTTTC
eIF2a183-R	TTGCTACCCTGAAACAGAACTTCCAGGCGCCTATTAATATTATTAATGAGTACTTCCCG
3CMBP-F	CTGGAAGTTCTGTTTCAGGGTAGCAAACTGGTAATCTGGATTAACGGCG GGTGATGTTTAAACTGGTCTAGAAAGCTTTTCAGTGATGGTG
MBPHis-R	GTGATGGTGGTGATGGTGGTGAGTCTGCGCGTCTTTCAGGGC

Pull-down assays

Streptavidin magnetic particles (Promega) were used in a pull-down assay for the analysis of the binding of the heterologously expressed eIF2α protein to GA. 2 nMol Biotinylated GA methylester **4b** (bGS) was incubated for 30 min at room temperature with 100 µg washed beads (volume 100 µl). Subsequently the beads were washed with PBS using a magnetic stand according to the manufacturer’s protocol (Promega). The charged beads were further incubated in 100 µl of 0.2 nMol eIF2α protein for another 30 min at room temperature, either

purified eIF2α (1-183) or eIF2α (1-302) fused to the MBP-His8 tag carrying a 3C-protease cleavage site, named R-C04 (63 kD) and R-C03 (77 kD), respectively. After removal of the unbound proteins, the beads were washed once with PBS. After this wash, proteins bound to the beads were analysed after boiling in 30 µl Laemmli Loading Buffer at 96°C for 5 minutes and separation on „Any kD“ SDS PAGE gels (Biorad). Gels were stained using „Instant Blue“ (Expedeon). Biotinylated protein A (bPA; Sigma) was used as a positive control for binding to the streptavidin magnetic beads. A binding curve with a range of 1, 2.5, 5; and 10 µg of protein A conjugate was made to determine the optimal condition for the binding assay. (Data not shown).

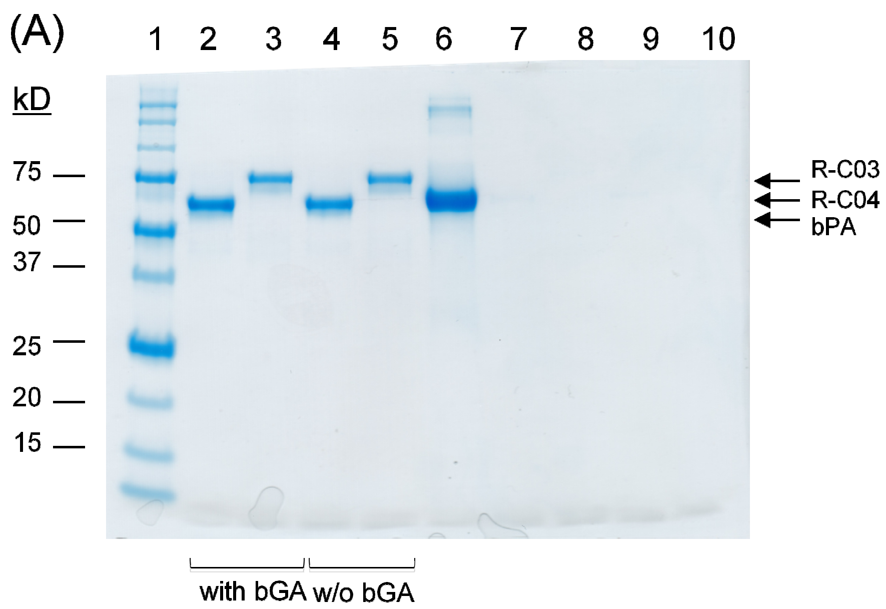

Lane	Sample	Amount
1	Precision Plus unstained (Biorad)	10 µl
2	A1 supernatant R-C03 (0.2 nM) + bGA (2 nM)	15 µl
3	B1 supernatant R-C04 (0.2 nM) + bGA (2 nM)	15 µl
4	C1 supernatant R-C03 (0.2 nM) w/o bGA	15 µl
5	C3 supernatant R-C04 (0.2 nM) w/o bGA	15 µl
6	D supernatant bPA (positive control)	15 µl
7	A1 wash R-C03 (0.2 nM) + bGA (2 nM)	15 µl
8	B1 wash R-C04 (0.2 nM) + bGA (2 nM)	15 µl
9	C1 wash R-C03 (0.2 nM) w/o bGA	15 µl
10	C3 wash R-C04 (0.2 nM) w/o bGA	15 µl

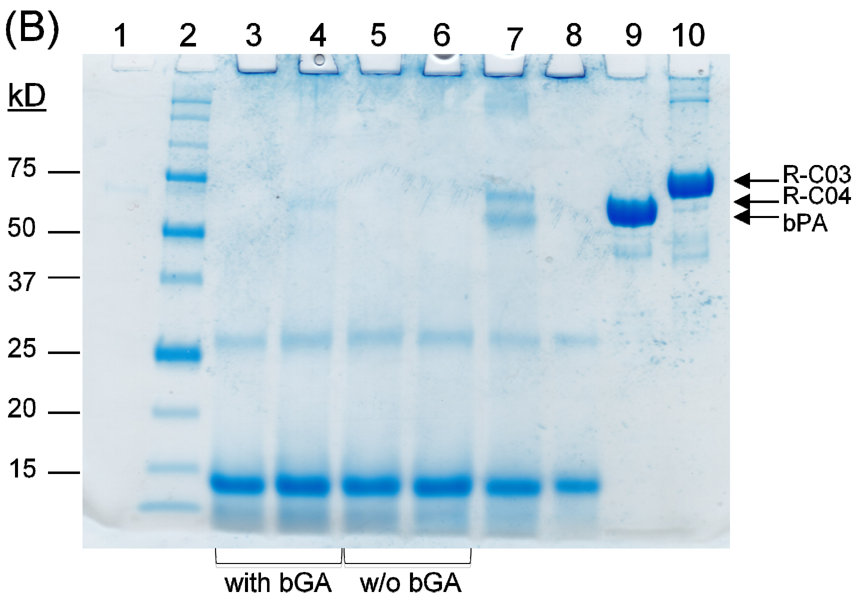

Lane	Sample	Amount
1	D wash bPA (positive control)	15 µl
2	Precision Plus unstained (Biorad)	10 µl
3	A1 beads R-C03 (0.2 nM) + bGA (2 nM)	15 µl
4	B1 beads R-C04 (0.2 nM) + bGA (2 nM)	15 µl
5	C1 beads R-C03 (0.2 nM) w/o bGA	15 µl
6	C3 beads R-C04 (0.2 nM) w/o bGA	15 µl
7	D beads bPA (positive control)	15 µl
8	Positive control beads only	15 µl
9	eIF2a_1-183_3C_MBP_His8 (R-C03)	5 µg
10	eIF2a_1-303_3C_MBP_His8 (R-C04)	5 µg

Figure. Pull-down asays with heterologously expressed eIF2α protein. (A) Lane 2 – 5 show the unbound fractions of eIF2α (R-C04 and R-C03) in the presence (lane 2 - 3) or in the absence of biotinylated GA methylester **4b** (bGA; lane 4 - 5). The non-bound BSA carrier protein of the biotinylated protein A (bPA) conjugate is shown in lane 6 but is not visible due to the high concentration of the BSA carrier protein, which masks the bPA. Lane 7-10 represent the wash fraction of the beads from the respective samples lanes 2 - 5. (B) Lane 3 - 6 show the bound protein to the magnetic streptavidin beads. The bound 50 kDa protein A (bPA) was visible in lane 7 (lower band) as well as some of the BSA carrier protein (upper band). Lane 9 and 10 show the quality of the purified protein eIF2α (R-C04 and R-C03).

For the binding competition experiments the purified human subunit of eIF2 was used, which was isolated using two steps of affinity chromatography. The human eIF2α purified by metal chelate binding to the His-tag and MBP binding showed highly purified fractions of this protein. There is no sequence information about the *Trichoplusia ni* eIF2 complex neither could we detect co-purification of the complex. Furthermore, there is nothing known whether the truncated human eIF2α or the full length protein is able to assemble into the insect eIF2 complex. Therefore we decided to evaluate the binding of GA-biontinylated first to the purified protein. The result of this experiment was that there is no binding detectable.

References:

1. Bleckmann M, Fritz MH, Bhuju S, Jarek M, Schurig M, Geffers R, Benes V, Besir H, van den Heuvel J. (2015). Genomic Analysis and Isolation of RNA Polymerase II Dependent Promoters from Spodoptera frugiperda. PLoS One *10*, e0132898.
2. Karste K, Bleckmann M, van den Heuvel J. (2017). Not Limited to E. coli: Versatile Expression Vectors for Mammalian Protein Expression. Methods Mol Biol *1586*, 313-324.
